# Challenges that early career psychiatrists can face on compulsory treatment

**DOI:** 10.1192/j.eurpsy.2024.138

**Published:** 2024-08-27

**Authors:** E. Chumakov

**Affiliations:** Department of Psychiatry and Addiction, St Petersburg State University, St Petersburg, Russian Federation

## Abstract

**Abstract:**

The delivery of mental health care worldwide often involves compulsory treatment, a practice encountered by early career psychiatrists from the outset of their training. Despite its prevalence, little research has explored the challenges faced by trainees and early career psychiatrists when compelled to administer treatment without patient’s consent. This presentation will synthesize research data and offer personal reflections on the author’s experiences.

Challenges that early career psychiatrists can face regarding compulsory treatment can be categorized into personal, professional, and institutional. Personal challenges encompass the emotional stress associated with applying coercive measures, coping with negative emotions, and managing service users’ attitudes toward treatment without consent. There is also concern that compulsory treatment may elevate the risk of emotional burnout. Professional challenges involve the administrative burden associated with organizing compulsory treatment, often exacerbated by the formalization of the process as a bureaucratic procedure in many European countries. Additionally, dealing with legal processes, including interactions with lawyers and courts, can pose significant difficulties, even though it is clearly done to protect the rights of the persons receiving care. Institutional challenges encompass the overall policy of providing compulsory psychiatric care in the psychiatrist’s home country and the specific practices of coercive measures in a given treatment facility. Furthermore, the lack of dedicated time for ethics of coercion during training is a common issue.

In the current landscape of mental health care, early career psychiatrists must undergo training to handle coercive measures. While these measures are sometimes unavoidable, ethical principles must guide their administration. Additionally, access to supervision/mentoring is crucial for early career professionals facing challenging cases.

**Disclosure of Interest:**

None Declared

